# Blockade of Cannabinoid CB1 Receptors in the Dorsal Periaqueductal Gray Unmasks the Antinociceptive Effect of Local Injections of Anandamide in Mice

**DOI:** 10.3389/fphar.2017.00695

**Published:** 2017-10-04

**Authors:** Diego C. Mascarenhas, Karina S. Gomes, Tatiani Sorregotti, Ricardo L. Nunes-de-Souza

**Affiliations:** ^1^Joint Graduate Program in Physiological Sciences, Federal University of São Carlos and São Paulo State University, São Carlos, Brazil; ^2^Laboratory of Neuropsychopharmacology, School of Pharmaceutical Sciences, São Paulo State University, Araraquara, Brazil

**Keywords:** vanilloid substrates, cannabinoid substrates, anandamide, periaqueductal gray, antinociception

## Abstract

Divergent results in pain management account for the growing number of studies aiming at elucidating the pharmacology of the endocannabinoid/endovanilloid anandamide (AEA) within several pain-related brain structures. For instance, the stimulation of both Transient Receptor Potential Vanilloid type 1 (TRPV1) and Cannabinoid type 1 (CB1) receptors led to paradoxical effects on nociception. Here, we attempted to propose a clear and reproducible methodology to achieve the antinociceptive effect of exogenous AEA within the dorsal periaqueductal gray (dPAG) of mice exposed to the tail-flick test. Accordingly, male Swiss mice received intra-dPAG injection of AEA (CB1/TRPV1 agonist), capsaicin (TRPV1 agonist), WIN (CB1 agonist), AM251 (CB1 antagonist), and 6-iodonordihydrocapsaicin (6-IODO) (TRPV1 selective antagonist) and their nociceptive response was assessed with the tail-flick test. In order to assess AEA effects on nociception specifically at vanilloid or cannabinoid (CB) substrates into the dPAG, mice underwent an intrinsically inactive dose of AM251 or 6-IODO followed by local AEA injections and were subjected to the same test. While intra-dPAG AEA did not change acute pain, local injections of capsaicin or WIN induced a marked TRPV1- and CB1-dependent antinociceptive effect, respectively. Regarding the role of AEA specifically at CB/vanilloid substrates, while the blockade of TRPV1 did not change the lack of effects of intra-dPAG AEA on nociception, local pre-treatment of AM251, a CB1 antagonist, led to a clear AEA-induced antinociception. It seems that the exogenous AEA-induced antinociception is unmasked when it selectively binds to vanilloid substrates, which might be useful to address acute pain in basic and perhaps clinical trials.

## Introduction

Pain-related diseases have been extensively investigated in order to unmask its complex neurobiology and underlying mechanisms, and/or to provide novel treatment options. In this context, several neurotransmitters have been implicated mediating nociception, for instance, opioids (Yaksh and Noueihed, 1985; Jensen and Yaksh, 1989; Cornelio and Nunes-de-Souza, 2009; Morgan et al., 2014), glutamate (Yaksh and Noueihed, 1985; Palazzo et al., 2013; Wilson-Poe et al., 2013), serotonin (Eschalier et al., 1989; Baptista-de-Souza et al., 2014; de Freitas et al., 2014), and endocannabinoids (Meng et al., 1998; Suplita et al., 2005; Olango et al., 2012). More recently, vanilloid compounds, which are known to activate the Transient Receptor Potential Vanilloid – type 1 (TRPV1) channels, emerged as an important neurotransmission system modulating nociception (e.g., McGaraughty et al., 2003; Starowicz et al., 2007; Mascarenhas et al., 2015).

The TRPV1 channels were found to be expressed in primary afferent neurons and implicated in transmitting noxious stimuli to the spinal cord [for a review, see Salat et al. (2013)]. Besides their role in pain modulation on peripheral nervous system (Gewehr et al., 2011), TRPV1 are also found in brainstem areas including the periaqueductal gray matter (PAG) (Cristino et al., 2006). This midbrain structure is highly involved in the modulation of defensive reactions such as fear/anxiety states as well as nociception (e.g., Starowicz et al., 2007; Lisboa and Guimaraes, 2012; Mascarenhas et al., 2013, 2015).

The midbrain PAG is part of the descending inhibitory system responsible for inhibiting pain processing at spinal cord level [for a review see Millan (2002)]. The PAG sends monosynaptically projections to the rostral ventromedial medulla (RVM) modulating *ON*- and *OFF*-cells that when activated are responsible, respectively, for facilitating and inhibiting pain at spinal cord level (Palazzo et al., 2008). This PAG–RVM circuitry expresses several neurotransmitters systems, e.g., glutamate (Palazzo et al., 2013), cannabinoid (CB; Hohmann et al., 2005), opioid (Wang and Wessendorf, 2002), vanilloid (Maione et al., 2006; Palazzo et al., 2008), and is known to be a pivotal supraspinal circuitry involved in the central modulation of pain (Jensen and Yaksh, 1989; Heinricher et al., 2009).

Several authors have investigated particularly the role of CBs and vanilloids in this circuitry modulating nociception. For instance, the stimulation of Cannabinoid receptor type 1 (CB1), expressed in both glutamatergic and GABAergic neurons in the PAG, leads to inhibition or excitation, respectively, of the pain modulating circuitry located in the RVM (Vaughan et al., 2000; Maione et al., 2006; Palazzo et al., 2008). Therefore, CB1-mediated nociception is under a complex modulation and paradoxical effects have been reported (Meng et al., 1998; Meng and Johansen, 2004; Maione et al., 2006). In addition, TRPV1 stimulation causes glutamate release within the RVM which in turn activates the descending inhibitory system, leading to antinociception (Palazzo et al., 2002; Starowicz et al., 2007; Mascarenhas et al., 2015). However, contrasting effects, i.e., hypernociception, have also been reported following TRPV1 stimulation, an action attributed to the capacity of TRPV1 desensitization (McGaraughty et al., 2003).

The investigation of the descending inhibitory system gained a novel impulse when vanilloid substrates showed to be closely related to the CB substrates. In this context, the two major endocannabinoids, *N*-arachidonoyl ethanolamine (anandamide; AEA) and 2-arachidonoyl glycerol (2-AG), have been implicated in the modulation of pain (Olango et al., 2012) and the former compound is known to bind to both CB1 and TRPV1 channels (Zygmunt et al., 1999; Ross et al., 2001; Maione et al., 2006). Additionally, Maione et al. (2006) showed that the inhibition of AEA degrading fatty acid amide hydrolase (FAAH) enzyme in the ventrolateral PAG (vlPAG) provoked either antinociception or pronociception via TRPV1 or CB1 activation, respectively, in rats subjected to the plantar test. However, it remains to be determined whether exogenous AEA injected into the PAG plays a role in the modulation of acute pain.

Since CB and vanilloid substrates lead to paradoxical effects on nociception due to physiological (different neurons population expressing CB1 receptors) and pharmacological (desensitization phenomenon) properties of each system, respectively, we hypothesized whether exogenous AEA might lead to a more clear and reproducible effect on nociception according to the substrates recruited within the mouse dorsal periaqueductal gray (dPAG). Thus, this study sought to demonstrate the role of exogenous AEA acting specifically either at TRPV1 or CB1 receptors located within the dPAG in the modulation of the nociceptive response. To that end, firstly we investigated the effects of intra-dPAG injections of AEA, capsaicin (a TRPV1 agonist), WIN 55,212-2 (a CB1 agonist), AM251 (a CB1 receptor antagonist), or 6-iodonordihydrocapsaicin (6-IODO) (a TRPV1 antagonist) on acute nociceptive response assessed through the tail-flick test (Experiments 1A–E). Then, the effects of intra-dPAG AEA on nociception were investigated under local blockade of CB1 (Exp. 2) or TRPV1 (Exp. 3) receptors.

## Materials and Methods

### Animals

Subjects were 181 male Swiss adult mice (UNESP – Universidade Estadual Paulista, São Paulo, Brazil), weighing 28–35 g at testing. They were housed in groups of 10 per cage (41 cm × 34 cm × 16 cm) and maintained under a normal 12 h light cycle (lights on 07:00 h) in a temperature controlled environment (23 ± 1°C). Food and water were freely available except during the brief test periods. All mice were naïve at the beginning of experiments and each mouse was used once. All efforts were made to minimize animal suffering.

### Drugs

The drugs were capsaicin (0.01, 0.1, or 1 nmol), a TRPV1 agonist, 6-IODO (1 or 3 nmol), a TRPV1 antagonist and (*R*)-(+)-[2,3-dihydro-5-methyl-3-(4-morpholinylmethyl)pyrrolo[1,2,3-*de*]-1,4-benzoxazin-6-yl]-1-naphthalenylmethanone mesylate (WIN – 1, 10, or 50 nmol), a CB1 agonist, dissolved in undiluted dimethylsulfoxide (DMSO) due to solubility issues. Given that lipids in Tocrisolve^TM^ (a formulation composed of a 1:4 ratio of soya oil/water which is emulsified with the block co-polymer, Pluronic F68) can be conveniently diluted with any aqueous medium for further use, AEA (CB1/TRPV1 agonist) which is already sold in Tocrisolve^TM^ (AEA; 0.5, 5.0, or 50 pmol) was diluted in saline solution (NaCl 0.9%). Lastly *N*-(piperidin-1-yl)-5-(4-iodophenyl)-1-(2,4-dichlorophenyl)-4-methyl-1H-pyrazole-3-carboxamide (AM251 – 1 or 10 pmol), a CB1 antagonist, was dissolved in DMSO 20% in saline (0.9% NaCl). Undiluted DMSO, saline, and DMSO 20% were used as vehicles for their respective groups. An additional group treated with Tocrisolve^TM^ was used for a comparison with other vehicle groups. Capsaicin, AEA, Tocrisolve^TM^, 6-IODO, and WIN were purchased from Tocris Cookson, Ballwin, MO, United States and AM251 from Sigma–Aldrich. The doses were based in pilot and previous studies (Maione et al., 2006; Moreira et al., 2007; Mascarenhas et al., 2013, 2015; Batista et al., 2015). The mass weight of each drug necessary for samples of 25 μL in the doses described were as follow: 50 nmol AEA = 2.12 mg; 10 nmol capsaicin = 3.75 mg; 50 nmol WIN = 3.25 mg; 10 nmol AM251 = 6.94 mg; and 3 nmol 6-IODO = 1.57 mg. Evidently, all drugs had to be diluted from this first solution to reach the proper doses. The final microinjection volume necessary to deliver the referred doses into the dPAG was 0.2 μL.

### Surgery and Microinjection

Mice received a Stereotaxic (Kopf Instruments) unilateral implant of a 7 mm stainless steel guide cannula (26-gauge; Insight Equipamentos Cientificos Ltda.) targeted to the dPAG under ketamine + xylazine anesthesia (100 and 10 mg/kg, i.p.). The guide cannula was fixed to the skull using dental acrylic and jeweler’s screws. Stereotaxic coordinates (Paxinos and Franklin, 2004) for the dPAG (dorsolateral and dorsomedial columns) were 4.1 mm posterior to bregma, 1.4 mm lateral to the midline, and 2.3 mm ventral to the skull surface, with the guide cannula angled 26° to the vertical. A dummy cannula (33-gauge stainless steel wire; Fishtex Industry and Commerce of plastics Ltd.), inserted into each guide-cannula immediately after surgery, served to reduce the incidence of occlusion. At the end of the stereotaxic surgery, each mouse received an intramuscular injection of penicillin-G benzathine (Pentabiotic, 56.7 mg/kg in a 0.1 mL volume; Fort Dodge, Campinas, São Paulo, Brazil) and a subcutaneous injection of the anti-inflammatory analgesic Banamine (3.5 mg/kg flunixin meglumine, Intervet Schering-Plough, Rio de Janeiro, RJ, Brazil, in a volume of 0.3 mL).

Five to seven days after surgical recovery and clearance of post-operative anti-inflammatory drugs, solutions were injected into the dPAG, blind to treatment, by microinjection units (33-gauge stainless steel cannula; Insight Equipamentos Cientificos Ltda.), which extended 1.0 mm beyond the tips of the guide cannula. Each microinjection unit was attached to a 2 μL Hamilton microsyringe via polyethylene (PE-10) tubing, and administration was controlled by the experimenter at a rate of 0.2 μL (volume injected) over a period of approximately 20 s. The microinjection procedure consisted of gently restraining the animal, removing off the dummy cannula, inserting the injection unit, infusing the solution, and keeping the injection unit *in situ* for further 60 s. Confirmation of successful infusion was obtained by monitoring the movement of a small air bubble in the PE-10 tubing.

### Apparatus; Tail-Flick Test

**FIGURE 1:**
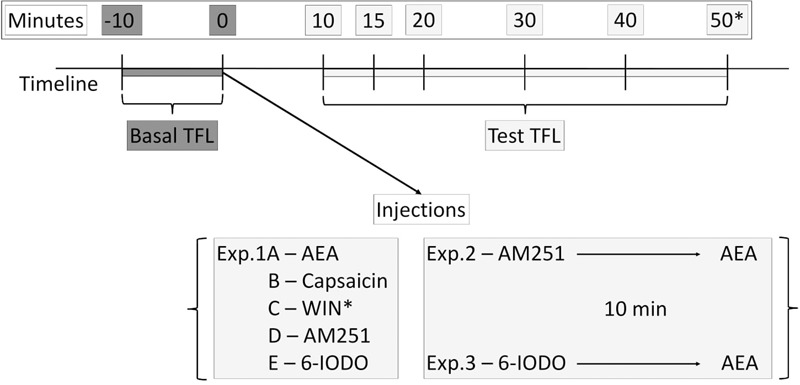
Timeline of the tail-flick test showing basal TFLs (dark gray boxes) and test TFLs (light gray boxes) recordings as well as injection procedures performed at the Exps. 1–3.

Nociception was assessed using the tail-flick test as previously described (Siegfried et al., 1987). To measure tail-flick latency (TFL), each mouse was gently restrained and the light source was focused on the distal portion of the mouse tail. A deflection of the tail activated a photocell mounted above it and terminated test. The light intensity was adjusted to 45 μA to obtain baselines from 2.0 to 3.0 s. Selection of the light intensity was based on pilot studies and it was kept constant throughout the Experiments. A cut-off time of 6 s was used in nonreactive animals. Tail-flick latencies were recorded 0 and 10 min before and 10, 15, 20, 30, and 40 min after pharmacological treatment into mice dPAG. In Exp. 1C at 50 min it was necessary an additional TFL recording due to a delay WIN-induced antinociception. In Experiments 2 and 3, pretreatment and treatment occurred within a time interval of 10 min (**Figure 1**). A pilot study was carried out aiming at revealing whether seven (or eight in the case of Exp. 1C) TFL records were able to induce tissue damage and no apparent effect was observed up to 24 h later the last measure. Each TFL was normalized by calculating an analgesia index (AI):

(1) $$\mathrm{AI}\;=\;\frac{\left(\mathrm{test}\;\mathrm{TFL})\;-\;(\mathrm{average}\;\mathrm{baseline}\;\mathrm{TFL}\right)}{6\;-\;(\mathrm{average}\;\mathrm{baseline}\;\mathrm{TFL})},$$

where AI = analgesia index; test TFL = latency of tail withdrawal scored 10, 15, 20, 30, and 40 (50 min in Exp. 1C) min after pharmacological treatment; average baseline TFL = average of the basal latencies of tail withdrawal 10 and 0 min before pharmacological treatment; 6 = cut-off time in seconds.

### Procedures

All healthy animals were transported to the experimental room and left undisturbed for at least 1 h for habituation before Experiments commence.

#### Experiments 1A–E: Intra-dPAG Injections of AEA, Capsaicin, WIN, AM251, or 6-IODO on Nociception in Mice

On test day, mice had two baseline TFLs recorded, at an interval of 10 min, and subsequently underwent intra-dPAG injections of AEA (vehicle, 0.5, 5.0, or 50 pmol; Exp. 1A), capsaicin (vehicle, 0.01, 0.1, or 1 nmol; Exp. 1B), WIN (vehicle, 1, 10, or 50 nmol; Exp. 1C), AM251 (vehicle, 1 or 10 pmol; Exp. 1D), or 6-IODO (vehicle, 1 or 3 nmol; Exp. 1E). Further TFLs were carried out at 10, 15, 20, 30, and 40 min after intra-dPAG microinjection of the solutions. In Exp. 1C, a further TFL record at 50 min post-treatment was also performed.

#### Experiments 2 and 3: Assessment of Intra-dPAG AEA Effects under Local Blockade of CB1 or TRPV1 Receptors in Mice Nociception

Aiming at revealing AEA effects specifically at vanilloid or CB substrates, mice had two baseline TFLs recorded, as described for Exps. 1A–E, following intra-dPAG administration of AM251 (10 pmol; Exp. 2) or 6-IODO (1 pmol, Exp. 3) at intrinsically inactive doses on nociception. Ten minutes later, they received local injections of AEA (vehicle, 0.5, 5.0, or 50 pmol). Animals were then subjected to the tail-flick test at 10, 15, 20, 30, and 40 min after the second microinjection.

### Histology

At the end of testing, all animals received an intra-dPAG 0.2 μL infusion of 1% Evans blue, according to the microinjection procedure described in the Section “Surgery and Microinjection.” The animals were then sacrificed in a CO_2_ chamber, their brains removed and injection sites histologically verified through coronal sections performed with a cryostat (Leica CM 1850) and a microscope (Leica DMLB) according to the atlas of Paxinos and Franklin (2004).

### Data Analysis

Data were subjected to Levene’s test of homogeneity followed by two-way analysis of variance [ANOVA; factor 1: treatment; factor 2: time (repeated measures)]. When appropriate data were subjected to the Duncan’s Multiple Comparisons Test. A value of *P* ≤ 0.05 was set for significance.

### Ethics Statement

This study was carried out in accordance with the recommendations of the Brazilian Society of Science of Laboratory Animals (SBCAL), which complies with international guidelines for animal use and welfare. The protocol was approved by the local Research Ethics Committee (CEP/FCF/Car, Universidade Estadual Paulista, resolution 16/2013).

## Results

Firstly, given the different vehicles used to dissolve the drugs tested throughout the study, a comparison of the TFL of vehicle-treated mice was performed in order to exclude/detect any vehicle-mediated effects on nociception. The procedure was similar to that performed on Exps. 1A–E. Vehicle groups were saline (Exp. 1A), undiluted DMSO (Exps. 1B,C,E), and DMSO 20% in saline (Exp. 1D). Moreover, a Tocrisolve^TM^-treated group (*n* = 7) of animals within the dPAG was also included in this analysis since AEA, a lipid compound, must be dissolved in this formulation which allows a conveniently dilution in any aqueous medium. Importantly, two-way ANOVA did not reveal significance for any factor (all *F*-values ≤ 0.82; *P* > 0.05) (data not shown).

### Histology

**FIGURE 2:**
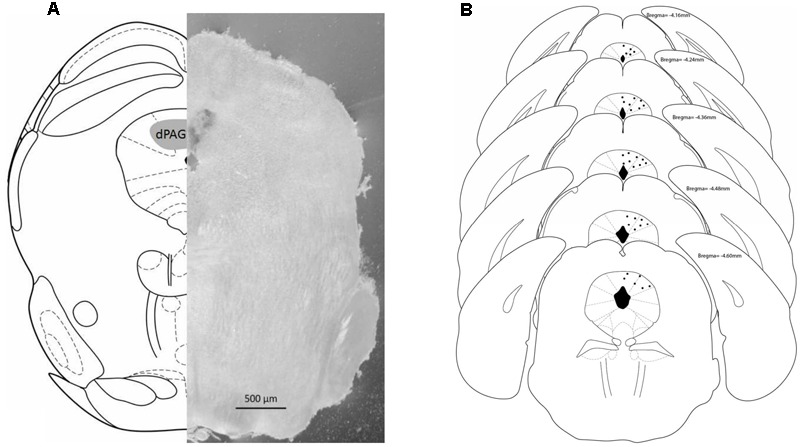
**(A)** Schematic representation (left) and photomicrograph (right) of the mouse brain. Both frames (left and right) correspond to –4.16 mm from bregma. **(B)** Schematic representation of microinjections sites within dPAG. Number of dots are representative and fewer than the actual number of animals due to overlapping. Histology revealed that all positive injection sites were between –4.16 and –4.60 mm from bregma (based on Paxinos and Franklin, 2004).

**Figure 2A** shows a schematic representation of a coronal section of the mouse brain (left) based on the atlas of Paxinos and Franklin (2004) highlighting the dorsal PAG (gray area) mirrored to a coronal photomicrograph (right) of a representative subject with a microinfusion site within the dPAG. **Figure 2B** also shows a schematic representation of brain sections indicating the microinfusion sites within the midbrain dPAG.

Histology confirmed that 141 mice had accurate cannula placements in the dPAG. Eighteen animals were used to investigate the effects of intra-dPAG AEA microinjection [Experiment 1A: vehicle (*n* = 5); AEA 0.5 pmol (*n* = 4); AEA 5.0 pmol (*n* = 4); AEA 50.0 pmol (*n* = 5)]. Twenty-two animals were used to assess the effects of capsaicin microinjections into the dPAG [Experiment 1B: vehicle (*n* = 6); cpsa 0.01 nmol (*n* = 5); cpsa 0.1 nmol (*n* = 6); cpsa 1 nmol (*n* = 5)]. Twenty-four animals were necessary to reveal the effects of intra-dPAG injections of WIN [Experiment 1C: vehicle (*n* = 7); WIN 1 nmol (*n* = 5); WIN 10 nmol (*n* = 6); WIN 50 nmol (*n* = 6)]; 13 animals were used in Experiment 1D [vehicle (*n* = 5); AM251 1 pmol (*n* = 4); AM251 10 pmol (*n* = 4);]. In Experiment 1E, 15 animals were necessary to reveal the intra-dPAG TRPV1 antagonism profile [vehicle (*n* = 4); 6-IODO 1 nmol (*n* = 6); 6-IODO 3 nmol (*n* = 5)]. Twenty-two animals were required to reveal the effects of AEA acting specifically on vanilloid substrates [Experiment 2: AM251–vehicle (*n* = 4); AM251–AEA 0.5 pmol (*n* = 6); AM251–AEA 5.0 pmol (*n* = 5); AM251–AEA 50 pmol (*n* = 7)]. Finally, 20 animals were used to reveal the opposite, i.e., AEA acting specifically on CB substrates [Experiment 3: 6-IODO–vehicle (*n* = 5); 6-IODO–AEA 0.5 pmol (*n* = 4); 6-IODO–AEA 5.0 pmol (*n* = 5); 6-IODO–AEA 50 pmol (*n* = 6)].

Additionally, seven mice that received a Trocisolve^TM^ injection into the dPAG were included in a separated group to be compared to the other vehicle-treated mice (see vehicle groups above).

Forty animals were excluded from the study. Eight of them had their baseline TFLs reached the cut-off time (i.e., 6 s), 29 were off-targets and 3 were outliers according to extreme studentized deviate (ESD) test.

### Experiment 1A: Lack of Effect of Intra-dPAG AEA on Nociception

**FIGURE 3:**
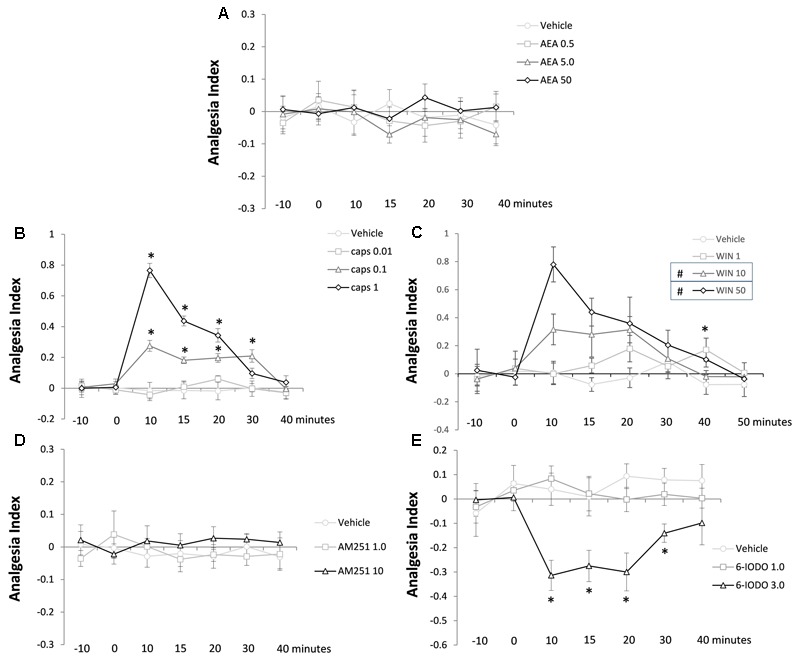
Lack of effects of AEA **(A)** and AM251 **(D)** and effects of capsaicin **(B)**, WIN **(C)**, and 6-IODO **(E)** injected into the dPAG on the TFL of mice exposed to the tail-flick test. Microinjection was performed at “zero” time. *N* = 4–7. Dots in the line chart represent mean ± SEM. Two-way ANOVA (repeated measures) followed by Duncan *post hoc* test. ^∗^*P* < 0.05 compared to vehicle-treated animals. ^#^*P* < 0.05 compared to vehicle-treated animals until 20 min post-treatment.

**Figure 3A** reveals the lack of effect of intra-dPAG AEA injections (0, 0.5, 5.0, or 50 pmol) on the TFL of mice recorded until 40 min post-injection. Two-way ANOVA did not reveal significance for any factor (all *F*-values ≤ 0.46; *P* > 0.05).

### Experiment 1B: Antinociceptive Effect of Intra-dPAG Capsaicin

**Figure 3B** shows the effects of intra-dPAG capsaicin microinjections (0, 0.01, 0.1, or 1 nmol) on the TFL of mice. Two-way ANOVA pointed out significance for treatment factor [*F*(3,18) = 36.60; *P* < 0.05], time factor [*F*(6,108) = 24.75; *P* < 0.05], and treatment × time interaction [*F*(18,108) = 13.78; *P* < 0.05]. Duncan’s test confirmed a dose-dependent antinociceptive effect of capsaicin (0.1 and 1 nmol) compared to vehicle-treated animals (*P* < 0.05). Animals presented a high magnitude antinociception when treated with capsaicin 1 nmol which lasted for 20 min. The intermediate dose (0.1 nmol) provoked a less intense, however, long lasting antinociceptive effect (up to 30 min) which was significantly lower than the higher dose until 20 min after treatment (*P* < 0.05). At the end of testing (40 min after treatment), animals no longer showed antinociception (*P* > 0.05).

### Experiment 1C: Antinociceptive Effect of Intra-dPAG WIN 55,212-2

Analyzing **Figure 3C** is possible to interpret the effects of intra-dPAG of WIN 55,212-2 (vehicle, 1, 10, or 50 nmol) on the TFL in mice. Two-way ANOVA showed significant effect of treatment [*F*(3,20) = 12.62; *P* < 0.05] and time [*F*(7,140) = 5.61; *P* < 0.05] factors as well as treatment × time interaction [*F*(21,140) = 2,34; *P* < 0.05]. *Post hoc* analysis confirmed a 20-min lasting antinociception on both 10 and 50 nmol-treated groups compared to vehicle-treated animals (*P* < 0.05). Sound with a dose-dependent effect the higher dose (50 nmol) of WIN provoked a more accentuated antinociceptive effect compared to the mild dose (10 nmol) at 10-min post-treatment (*P* < 0.05). The lower dose (1 nmol) of WIN caused a delayed antinociceptive effect at 40 min post-treatment compared to vehicle-treated animals (*P* < 0.05). At 50 min, animals no longer presented antinociception (*P* > 0.05).

### Experiment 1D: Lack of Effect of Intra-dPAG AM251 on Nociception

**Figure 3D** summarizes the lack of effects of intra-dPAG injections of AM251 (0, 1, or 10 pmol) on the TFL in mice. Two-way ANOVA did not reveal significance for any of the three factors (all *F*-values ≤ 0.83; *P* > 0.05).

### Experiment 1E: Hypernociceptive Effect of Intra-dPAG 6-IODO

**Figure 3E** reveals the effects of intra-dPAG 6-IODO (0, 1, or 3 nmol) on the TFL in mice. Two-way ANOVA pointed out significance effects for treatment factor [*F*(2,12) = 18.66; *P* < 0.05] and treatment × time interaction [*F*(12,72) = 2.34; *P* < 0.05] without revealing significance for time factor [*F*(6,72) = 1.32; *P* > 0.05]. Duncan’s *post hoc* test confirmed a 30-min lasting hypernociception in 6-IODO-tretated animals (3 nmol) compared to vehicle group. At 40 min after treatment, all animals had their TFL reached the baseline threshold (*P* < 0.05).

### Experiment 2: Antinociceptive Effect of Intra-dPAG AEA under Blockade of Local CB1 Receptors

**FIGURE 4:**
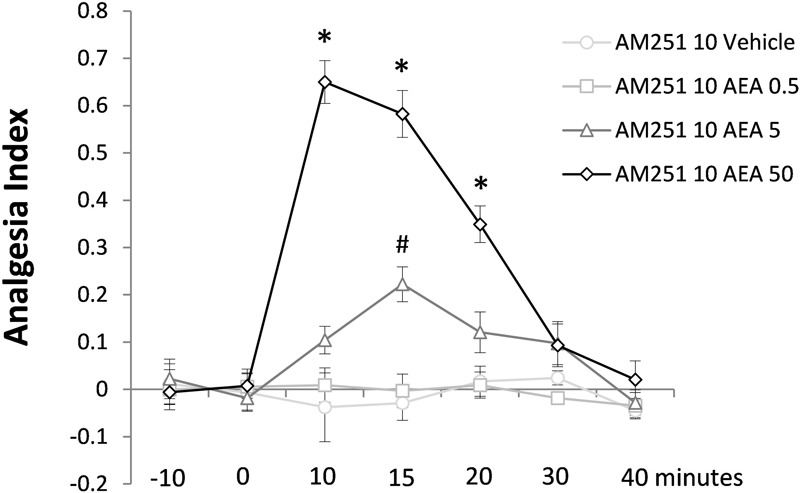
Antinociceptive effects of intra-dPAG AM251 microinjection followed by local AEA on the TFL of mice exposed to the tail-flick test. Microinjections were performed at “zero” time. *N* = 4–7. Dots in the line chart represent mean ± SEM. ^∗^*P* < 0.05 compared to vehicle-treated animals. Two-way ANOVA followed by Duncan *post hoc* test.

**Figure 4** shows the effect of intra-dPAG AEA (0, 0.5, 5.0, or 50 pmol) on nociceptive response of mice previously treated with AM251 (10 pmol, an intrinsically inactive dose; see Exp. 1C) into the same site. Two-way ANOVA revealed significant effects for all factors including their interaction; {treatment [*F*(3,18) = 77.91; *P* < 0.05]; time [*F*(6,108) = 20.06; *P* < 0.05]; treatment × time interaction [*F*(18,108) = 13.80; *P* < 0.05]}. Duncan’s multiple comparison test confirmed a dose-dependent antinociceptive effect of AEA (5.0 and 50 pmol) compared to vehicle-treated group. Similarly to the effects provoked by Capsaicin (Exp. 1B), 50 pmol AEA increased the AI of animals up to 20 min. However, at the dose of 5.0 pmol, AEA produced only a mild antinociceptive effect observed at 15 min followed intra-dPAG injection. Yet, none dose of AEA changed nociceptive response at 30 and 40 min after drug injection (*P* > 0.05).

### Experiment 3: Lack of Effect of Intra-dPAG AEA under Blockade of Local TRPV1 Receptors

**FIGURE 5:**
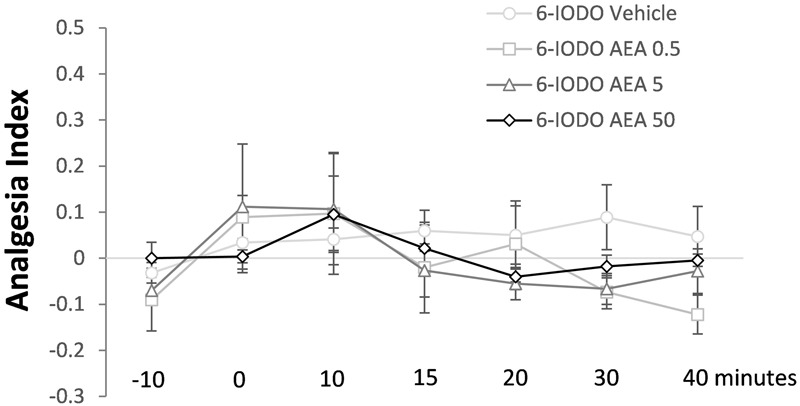
Lack of effects of intra-dPAG 6-IODO microinjection followed by local AEA on the TFL of mice exposed to the tail-flick test. Microinjections were performed at “zero” time. *N* = 4–6. Dots in the line chart represent mean ± SEM. Two-way ANOVA followed by Duncan *post hoc* test.

The last Experiment is summarized in **Figure 5** which shows lack of effects of intra-dPAG treatment of AEA (0, 0.5, 5.0, or 50 pmol) in animals previously treated with 6-IODO (1 nmol, an intrinsically inactive dose; see Exp. 1D) into the same site. Two-way ANOVA showed significant effects only for time factor [*F*(6,96) = 2.4; *P* < 0.05]. Duncan’s *post hoc* test confirmed a difference in the AI of the basal TFLs (-10 and 0 min) as well as in the -10 min TFL compared to 10 min TFL (*P* < 0.05).

## Discussion

The main results of this study point out that AEA (0.5–50 pmol), a TRPV1/CB1 agonist, injected into the mouse dPAG does not produce any intrinsic effect on acute pain as assessed through the tail-flick test (Exp. 1A). Conversely, intra-dPAG injections of capsaicin (0.1 and 1 nmol – Exp. 1B) or WIN (10 and 50 nmol – Exp. 1C) provoked a marked TRPV1- and CB1-dependent antinociception, respectively. While the blockade *per se* of CB1 receptors did not change nociceptive response (Exp. 1D), intra-dPAG injection of 6-IODO, a TRPV1 antagonist, produced a hypernociceptive effect (Exp. 1E). Interestingly, under blockade of CB1 receptors, intra-dPAG AEA produced a clear and consistent antinociceptive effect (Exp. 2). In contrast, the blockade of TRPV1 did not change the lack of effects of intra-dPAG injection of AEA on nociceptive response (Exp. 3).

Intra-dPAG injections of AEA (0.5–50 pmol), an endocannabinoid/endovanilloid agonist (Zygmunt et al., 1999; Van Der Stelt and Di Marzo, 2004; Marinelli et al., 2007), failed to alter TFL throughout the 40-min test. This unexpected result contrasts with previous findings showing that intra-vlPAG injections of mild doses of URB597 (an FAAH enzyme inhibitor), which in turn increases endogenous AEA, led to a vanilloid-mediated analgesia in rats exposed to the plantar test (Maione et al., 2006). However, those authors also reported that local low or high doses of the FAAH inhibitor provoked CB-mediated hyperalgesia (Maione et al., 2006). Based on present study, AEA acting specifically on CB substrates (Exp. 3) corroborated the lack of effect of AEA (Exp. 1A) suggesting that the doses of this vanilloid/CB agonist used in Exp. 1A might also have stimulated preferentially CB1 receptors. Indeed, the role of CB substrates on nociception is under a complex debate. A less likely possibility might account for this lack of effect considering a combined stimulation of TRPV1/CB1 receptors in Exp. 1, since it has been demonstrated a net null effect following both vanilloid/CB activation on the ventrolateral column of the PAG (Maione et al., 2006). In this regard, since previous results from our laboratory showed a vanilloid-mediated antinociception into the dorsal PAG (Mascarenhas et al., 2015), present study was conducted accordingly in the same midbrain column in an attempt to be consistent with previous findings. These site differences (dorsal versus ventrolateral columns) could also explain such discrepancies.

To clarify the involvement of dPAG TRPV1 and CB1 receptors in the modulation of the nociceptive response this study investigated the effects of specific vanilloid or CB agonists into the mouse dPAG on nociception. Interestingly, both capsaicin (0.1 and 1 nmol) and WIN (10 and 50 nmol) injected into the mouse dPAG increased dose-dependently the TFL of mice, suggesting a vanilloid- and a CB-dependent antinociception, respectively. Present study also reported a delayed CB-mediated antinociception following a local injection of a low dose (1 nmol) of WIN. In general, these results corroborate many other studies showing that capsaicin injected into various PAG columns provokes antinociception in rodents (Palazzo et al., 2002; Starowicz et al., 2007; Mascarenhas et al., 2015) possibly by facilitating the descending inhibitory system. Regarding CB1 role in the modulation of nociception, as presently shown with intra-dPAG injections of WIN, a great body of evidence have shown that phytocannabinoids (Lichtman and Martin, 1996; Meng et al., 1998), synthetic agonists (Meng et al., 1998), and endocannabinoids (Maione et al., 2006) induce antinociception as assessed through acute pain tests. However, contrasting results have also been reported with TRPV1 and CB1 manipulations. For instance, the hypernociception reported following vanilloid stimulation (McGaraughty et al., 2003) is an effect that has been attributed to the capacity of desensitization of TRPV1 receptors (Palazzo et al., 2008). In this later scenario, i.e., vanilloid-induced hypernociception, only high doses of TRPV1 agonist would be sufficient to induce desensitization (Palazzo et al., 2008). Regarding the hypernociceptive effects following CB1 receptor activation, previous studies have demonstrated immediate and delayed hypernocicetive effects when low doses of WIN or URB597 were injected into the rat vlPAG (Maione et al., 2006). This paradoxical profile of CB1 agonists seems to be due to the expression of CB1 receptors on both glutamatergic and GABAergic neurons at pain modulating circuitry of these murine species (Palazzo et al., 2008).

Accordingly, Exps. 1D and E were carried out in order to find intrinsically inactive doses of CB1 and TRPV1 antagonists on nociception when injected into the mouse dPAG. Exp. 1D revealed that AM251, a CB1 receptor antagonist, did not change nociceptive response of mice subjected to the tail-flick test, indicating lack of CB tonic control over dPAG CB1 receptors in the modulation of acute pain. This is in accordance to the fact that the CB system is recruited only on demand [for a review see Morena and Campolongo (2014) and Ulugol (2014)]. On the contrary, Exp. 1E showed that intra-dPAG 6-IODO (TRPV1 antagonist) at the highest dose (3 nmol) decreased the TFL, suggesting a hypernociceptive effect and therefore an endovanilloid tonus within the mouse dPAG modulating nociception. Starowicz et al. (2007) have first demonstrated a tonic endovanilloid facilitation of glutamate release within rats PAG, since 5′-iodoresiniferatoxin, a selective TRPV1 antagonist, facilitated nociceptive responses. In addition, authors have demonstrated that endovanilloids contribute to anxiety modulation. In this context, capsazepine (TRPV1 antagonist) injected into the PAG attenuated the defensive behavior of rats exposed to the elevated plus maze (Moreira et al., 2007).

The well-known CB1-mediated antinociception found in Exp. 1C seems to be due to the inhibition of GABA release from PAG interneurons, which in turn would contribute to disinhibition of PAG antinociceptive outputs (Moreau and Fields, 1986; Meng et al., 1998; Vaughan et al., 2000). It contrasts with the lack of effect of AEA acting specifically on CB substrates reported on Exps. 1A and 3. In this context, although no Experiment has assessed the said issue, it is likely that the doses of AEA stimulated both neurons population-expressing CB1 receptors and nociception might have been physiologically counterbalanced which accounts for the lack of effect. In addition, WIN displays different pharmacodynamics aspects (higher CB1 affinity therefore lower *K*i value) and it is not subjected to FAAH hydrolyses compared to AEA, which makes difficult the comparison of doses of a synthetic versus endogenous CB1 agonist. Furthermore, it is still necessary considerably more investigation to unravel the participation of glutamatergic and GABAergic neurons-expressing CB1 receptors since, physiologically, CB-mediated outcomes are under opposite pathways.

Attempting to determine the role of exogenous AEA binding preferentially at either TRPV1 or CB1 receptors, Exps. 2 and 3 consisted on evaluating nociceptive response in mice that had received intra-dPAG injection of AM251 (10 pmol) or 6-IODO (1 nmol) prior to local injections of AEA. Interestingly, contrasting with the results obtained in Exp. 1A, AEA (5.0 and 50 pmol) produced a marked antinociceptive effect only under the blockade of CB1 receptors (Exp. 2). AEA, at the highest dose, provoked a high-magnitude 20-min lasting antinociceptive effect possibly via vanilloid substrates, corroborating Exp. 1B, which revealed a similar capsaicin-induced antinociception. On the contrary, the blockade of vanilloid substrates failed to reveal any intrinsic effect of intra-dPAG AEA on nociception (Exp. 3), corroborating the lack of effect observed in Exp. 1A, where only AEA was injected. Thus, it seems reasonable to suggest that the doses of AEA used in Exp. 1A have also preferentially stimulated the CB substrates even though no antagonism was performed. In this context, in terms of pharmacodynamics, AEA binds to CB1 receptors with higher affinity (*K*i value between 37 and 116 nM) compared to TRPV1 receptor (*K*i value 1.66 μM) (Ross et al., 2001). Therefore, Experiment 1A seems to corroborate the higher affinity of AEA to CB substrates at a behavioral level since it revealed the same outcome of Exp. 3.

In this context, it has been shown that AEA might also act via other ligand-gated channels, such as the 5-HT3 and glycine receptors, which potentially could contribute to AEA-induced effects on nociception. However, the AEA role on 5-HT3 receptors highly depends on the abundance of this receptor at the cell surface of specific brain sites (Barann et al., 2002; Xiong et al., 2008). Furthermore, AEA seems to enhance glycine clearance in the synaptic cleft (Pearlman et al., 2003), which, physiologically, could impair the NMDA-dependent excitatory current and by extension AEA-induced antinociception which depends on glutamate release into RVM *OFF* cells. These evidence have weakened the concern with the potential influence of other channels in AEA effects together to the fact that several evidence point out the relevance of vanilloid/CB substrates in mediating AEA-induced effects (Zygmunt et al., 1999; Vaughan et al., 2000; Fenwick et al., 2017). In other words, these evidence strengthen the fact that Exps. 2 and 3 indeed led AEA to bind with specificity to vanilloid and CB substrates, respectively.

According to our hypothesis, AEA-induced antinociception seems to be easily reproduced when it is vectored to vanilloid substrates where only desensitization must be avoided. In other words, controlling the amount of endovanilloid that stimulates TRPV1 receptors, AEA will ultimate provoke antinociception. In contrast, aiming at studying CB1-mediated antinociception, one must take into account the complex neurophysiology of the CB substrates within the dPAG, where both glutamatergic and GABAergic neurons express CB1 receptors. In addition, it seems that while present results unmasks exogenous AEA antinociceptive effects after CB1 blockade in the PAG, FAAH inhibitors unravel endogenous AEA effects intra-vlPAG that might be pro- or antinociceptive depending on the doses (Maione et al., 2006).

Noteworthy, in Exp.1 the dosage of AEA (0.5–50 pmol) was a bit disconnected to that used for capsaicin (0.01–1 nmol). On the one hand, it might weaken our study in view of their different affinities for TRPV1 (AEA displays lower affinity to TRPV1 than capsaicin) and therefore a higher dosage of AEA and a lower dosage of capsaicin than those employed in the present study should have been considered. However, on the other hand, it strengthens the fact that a very low dose of AEA was able to induce antinociception via TRPV1 under local CB1 blockade (Exp. 2), corroborating the hypothesis that depending on the substrates (i.e., TRPV1 or CB1) AEA might provoke a clear and potent antinociceptive effect.

Finally, it is likely that a broader range of AEA doses on Exp. 3 would possibly reveal a different outcome of this compound acting preferentially on CB substrates, since the doses of AEA determine the proper CB1-mediated effects. However, analyzing the effects in Exp. 2, present data suggest an interesting approach to address exogenous AEA effects on nociception (i.e., vectoring AEA to act preferentially on vanilloid substrates) and eventually allowing promising clinical trials which until now does not seem to translate to humans the potential of CB compounds (e.g., AEA) in pain management.

As far as we know, present results are the first to demonstrate antinociceptive effects of exogenous AEA injected into the dPAG specifically in an acute pain test, i.e., tail-flick test. So far, only endogenous AEA has been investigated in the modulation of nociception. In conclusion, present study revealed an antinociceptive effect of exogenous AEA injected into the dPAG only when CB1 receptors were antagonized, suggesting an important role of AEA in the vanilloid substrates that modulate acute pain within this midbrain area of mice. Therefore, it makes relevant to investigate further approaches considering the role of AEA binding specifically in vanilloid substrates as a potential new methodology to address acute pain on basic research and perhaps clinical trials.

## Author Contributions

DM and RN-d-S designed the study. DM performed the acquisition, data analysis, and drafted the manuscript. DM, RN-d-S, KG and TS participated in interpretation of data and critically revised the manuscript for important intellectual content. DM, RN-d-S, KG and TS approved the manuscript in its final version.

## Conflict of Interest Statement

The authors declare that the research was conducted in the absence of any commercial or financial relationships that could be construed as a potential conflict of interest.
